# Genome Sequence of *Listeria monocytogenes* Strain HPB2088 (Serotype 1/2a), an Environmental Isolate Collected in Canada in 1994

**DOI:** 10.1128/genomeA.00760-16

**Published:** 2016-08-04

**Authors:** Arthur W. Pightling, Hugh Rand, Errol Strain, Franco Pagotto

**Affiliations:** a Listeriosis Reference Centre Microbiology Research Division, Bureau of Microbial Hazards, Food Directorate, Health Canada, Ottawa, Ontario, Canada; b Biostatistics and Bioinformatics Branch, Office of Analytics and Outreach, Center for Food Safety and Applied Nutrition, U.S. Food and Drug Administration, College Park, Maryland, USA

## Abstract

*Listeria monocytogenes* is a foodborne pathogen that causes severe illness. Thus, ongoing efforts at real-time whole-genome sequencing are of utmost importance. However, it is also important that retrospective analyses that place these data into context be performed. Here, we present the genome sequence of strain HPB2088, which was collected in 1994.

## GENOME ANNOUNCEMENT

*Listeria monocytogenes*, the etiologic agent of listeriosis, is a Gram-positive bacterium commonly found in plant, soil, and surface water habitats (1). Listeriosis is a life-threatening illness that may occur when foods contaminated with *L. monocytogenes* are consumed, especially by immunocompromised individuals such as neonates and elderly people (2). As part of ongoing efforts to minimize the impacts of listeriosis, governmental organizations routinely collect *L. monocytogenes* from foods and food-processing facilities and sequence their genomes in real time (prospective studies). In addition, groups are sequencing the genomes of historical *L. monocytogenes* isolates (retrospective studies). Here, we present the genome sequence of the Canadian *L. monocytogenes* strain HPB2088 (serotype 1/2a) collected in 1994 (NCBI BioSample no. SAMN02867555; https://www.ncbi.nlm.nih.gov/biosample/SAMN02867555). Studying HPB2088 may provide valuable insight into the biology and history of *L. monocytogenes* in Canada.

We assembled Illumina sequencing reads with SPAdes v3.0.0 (3), using the BayesHammer error correction tool (4). The assembly yielded 38 nonoverlapping contiguous sequences with 192.33-fold coverage, a total length of 2,979,080 nucleotides, and an *N*_50_ of 211,340 nucleotides (NCBI RefSeq assembly accession GCF_000712425.1; https://www.ncbi.nlm.nih.gov/assembly/GCF_000712425.1). The NCBI Prokaryotic Genome Annotation Pipeline was used to annotate the genome (5). A total of 2,994 features were identified, including 2,912 protein-coding regions, 15 pseudogenes, one CRISPR array, three rRNAs, 62 tRNAs, and one ncRNA. We also identified *Listeria* genomic island 1 (LGI1), a feature that, to date, has only been reported in a small group of Canadian isolates (6, 7).

Analysis of HPB2088 with pulsed-field gel electrophoresis indicates an LMACI.0001 AscI restriction digest pattern and an LMAAI.0001 ApaI pattern. The ribotype pattern is 21-S-4, or DUP-1045. During a previous study, the sequence type was determined with *in silico* multilocus sequence typing to be ST120 (*abcA* − 5, *bglA* − 6, *cat* − 2, *dapE* − 29, *dat* − 5, *ldh* − 3, *lhkA* − 1) (8). HPB2088 is a member of evolutionary lineage II and clonal complex CC8.

### Nucleotide sequence accession numbers.

This whole-genome shotgun project was deposited at DDBJ/EMBL/GenBank under the accession number JOKU00000000. The version described in this paper is the first version, JOKU01000000.
